# Supplementary material for: Systematically developing a registry of splice-site creating variants utilizing massive publicly available transcriptome sequence data
Source: Nat Commun. 2025 Jan 9;16:426. doi: 10.1038/s41467-024-55185-y (PMC11718197; doi:10.1038/s41467-024-55185-y)
Supplement: Supplementary file 2 — Description of Additional Supplementary Files [file 41467_2024_55185_MOESM2_ESM.pdf]

Supplementary Data Legend

Supplementary Data 1: Splice-site creating variants detected from transcriptome sequencing data of 1000 Genomes Project and their validation summary.

Supplementary Data 2: List of TCGA samples used in this study and the frequencies of their SSCVs, and matched WGS availability.

Supplementary Data 3: List of splice-site creating variants affecting cancer-related genes (defined in PanCanAtlas, Bailey et al., Cell, 2018) identified in TCGA transcriptome data.

Supplementary Data 4: List of splice-site creating variants overlapping Alu elements (>= 200-bp) and their summaries.

Supplementary Data 5: List of samples with novel NOTCH1 gain-of-function splice-site creating variants (c.5048-132G>C, c.5048-132G>T).

Supplementary Data 6: List of PCR primers used for validation of splice-site creating variants.

Supplementary Data 7: sgRNA and donor sequences used in genome editing via CRISPR-Cas9.

Supplementary Data 8: Antibodies information used in western blot analysis.

Supplementary Data 9: Summary of splice-site switching antisense oligonucleotides sequences used in this study.
