# Supplementary figures and images for: Systematically developing a registry of splice-site creating variants utilizing massive publicly available transcriptome sequence data
Source: Nat Commun. 2025 Jan 9;16:426. doi: 10.1038/s41467-024-55185-y (PMC11718197; doi:10.1038/s41467-024-55185-y)

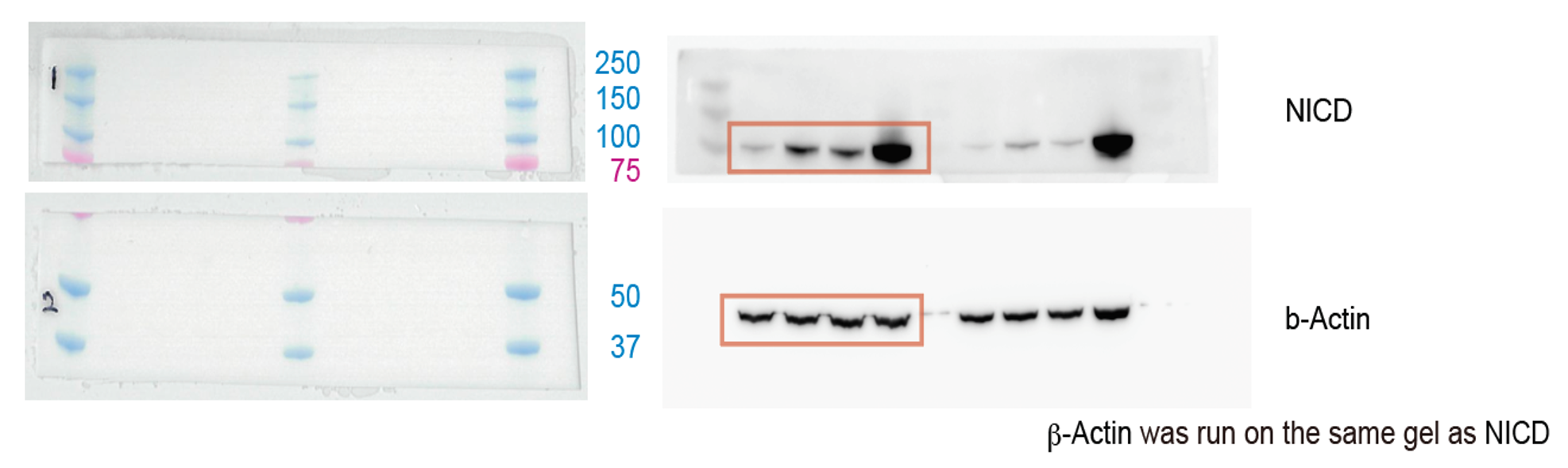

Supplement: Supplementary file 6 — Source Data [file 41467_2024_55185_MOESM6_ESM.zip › SourceData/Figure6d_source.png]

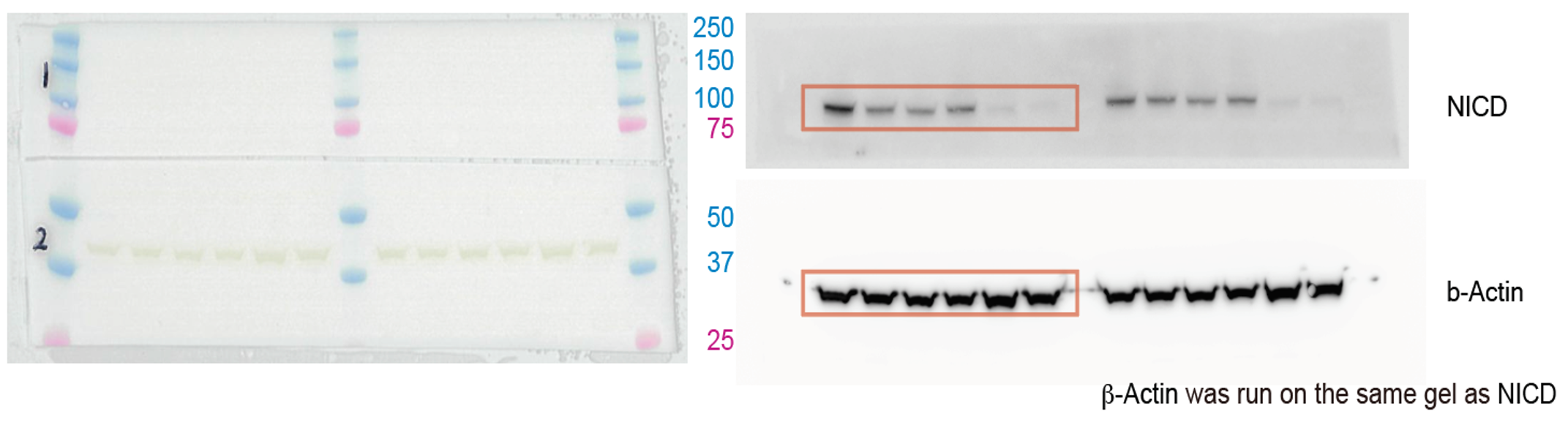

Supplement: Supplementary file 6 — Source Data [file 41467_2024_55185_MOESM6_ESM.zip › SourceData/Figure6f_source.png]

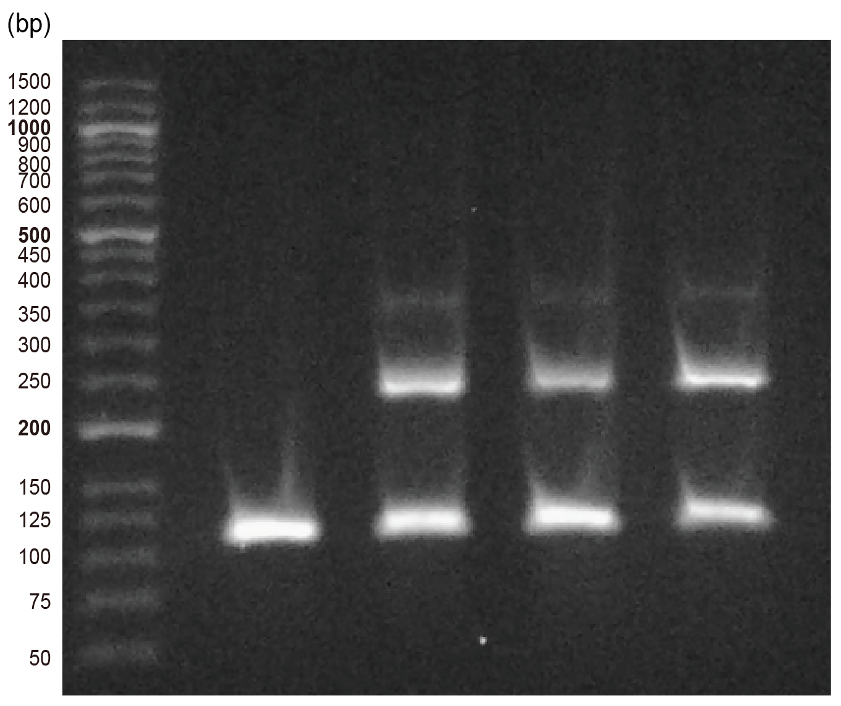

Supplement: Supplementary file 6 — Source Data [file 41467_2024_55185_MOESM6_ESM.zip › SourceData/Figure6c_source.png]

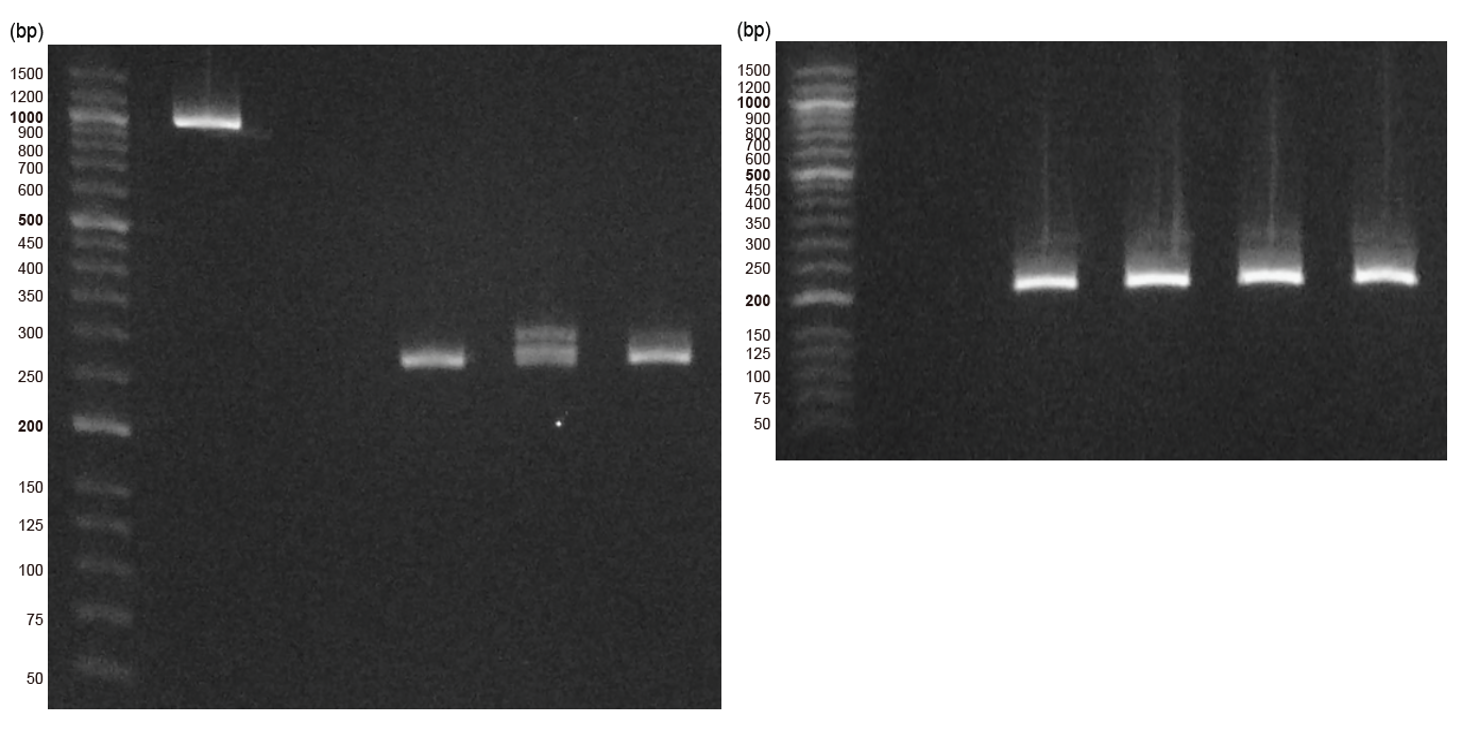

Supplement: Supplementary file 6 — Source Data [file 41467_2024_55185_MOESM6_ESM.zip › SourceData/SupplementaryFigure8b_source.png]

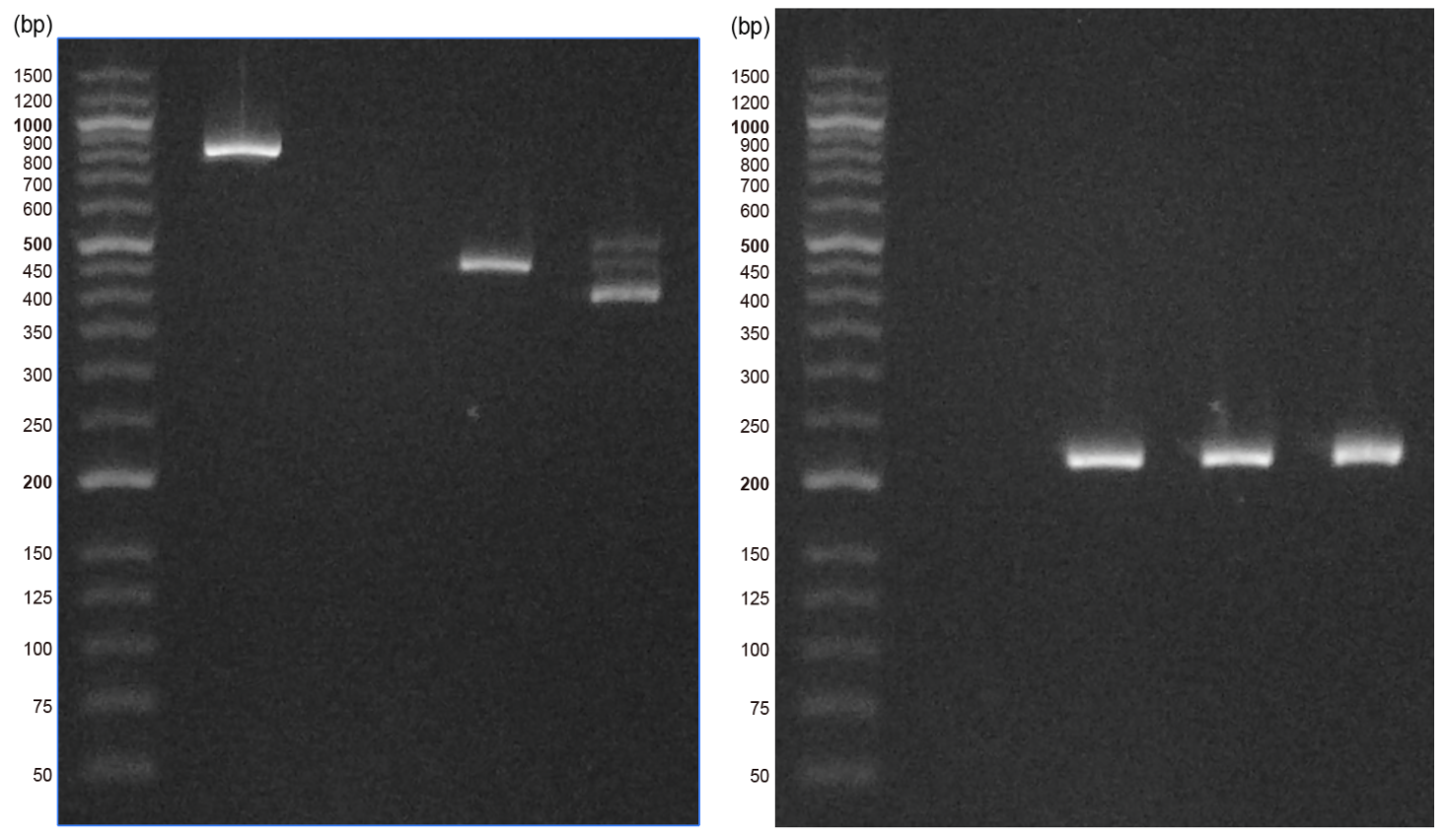

Supplement: Supplementary file 6 — Source Data [file 41467_2024_55185_MOESM6_ESM.zip › SourceData/SupplementaryFigure8a_source.png]

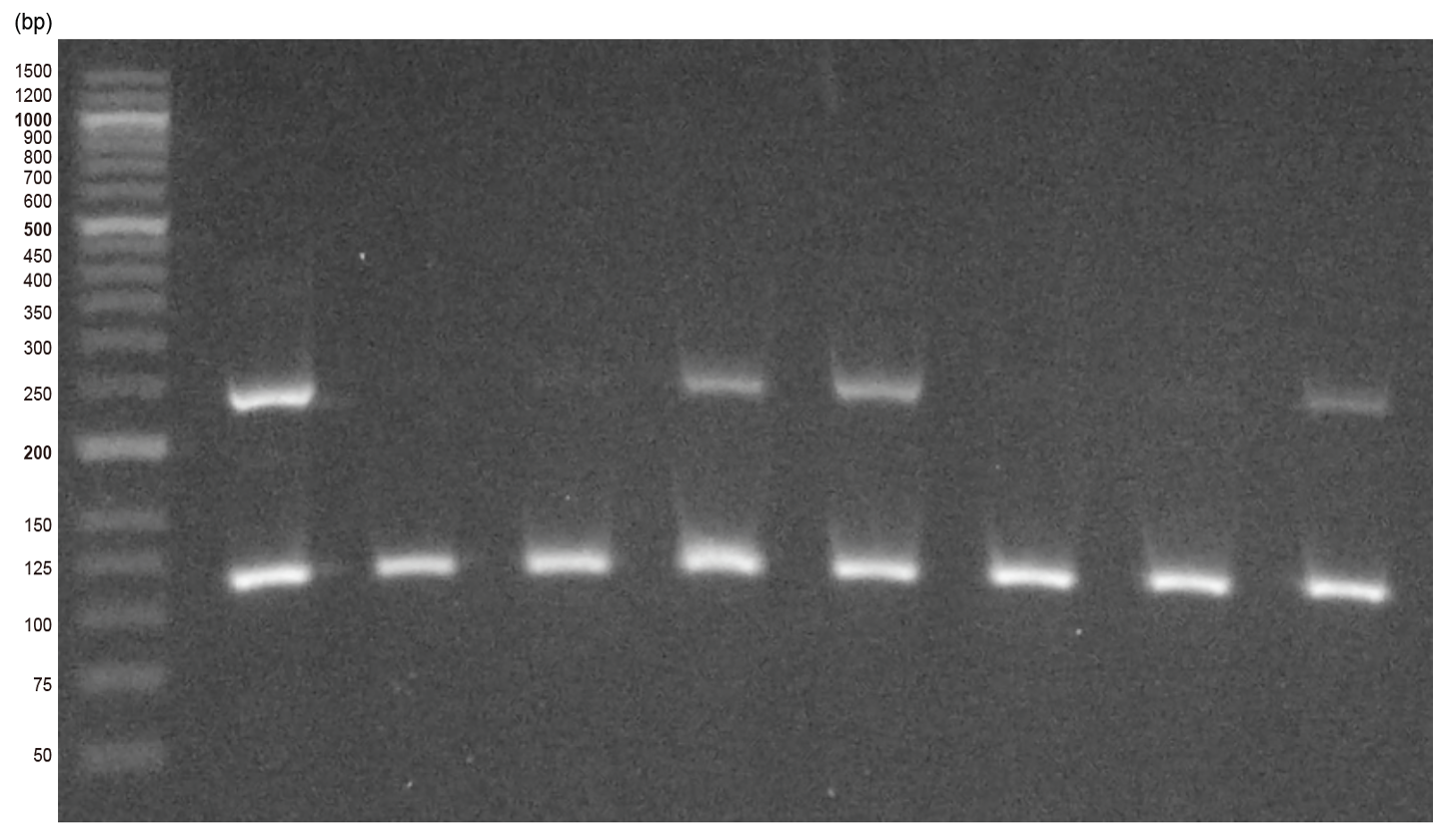

Supplement: Supplementary file 6 — Source Data [file 41467_2024_55185_MOESM6_ESM.zip › SourceData/Figure6e_source.png]
